# Evolutionary Dead End in the Galápagos: Divergence of Sexual Signals in the Rarest of Darwin's Finches

**DOI:** 10.1371/journal.pone.0011191

**Published:** 2010-06-23

**Authors:** Henrik Brumm, Heather Farrington, Kenneth Petren, Birgit Fessl

**Affiliations:** 1 Communication and Social Behaviour Group, Max Planck Institute for Ornithology, Seewiesen, Germany; 2 Department of Biological Sciences, University of Cincinnati, Cincinnati, Ohio, United States of America; 3 Charles Darwin Research Station, Puerto Ayora, Santa Cruz, Galápagos, Ecuador; 4 Durrell Wildlife Conservation Trust, Jersey, Channel Islands, United Kingdom; University of Sussex, United Kingdom

## Abstract

Understanding the mechanisms underlying speciation remains a challenge in evolutionary biology. The adaptive radiation of Darwin's finches is a prime example of species formation, and their study has revealed many important insights into evolutionary processes. Here, we report striking differences in mating signals (songs), morphology and genetics between the two remnant populations of Darwin's mangrove finch *Camarhynchus heliobates*, one of the rarest species in the world. We also show that territorial males exhibited strong discrimination of sexual signals by locality: in response to foreign songs, males responded weaker than to songs from their own population. Female responses were infrequent and weak but gave approximately similar results. Our findings not only suggest speciation in the mangrove finch, thereby providing strong support for the central role of sexual signals during speciation, but they have also implications for the conservation of this iconic bird. If speciation is complete, the eastern species will face imminent extinction, because it has a population size of only 5–10 individuals.

## Introduction

Speciation is the fundamental evolutionary process that generates biological diversity. New species originate and remain thereafter separate if they are reproductively isolated, in other words, reproductive isolation separates evolving lineages by cutting off gene flow between them [1]. Such reproductive isolation may be prezygotic, i.e. before fertilization, or postzygotic, i.e. after fertilization. Prezygotic reproductive isolation occurs when individuals do not mate because of behavioural differences, most importantly mate choice [2]. A premating barrier is often related to geographic variation in mating signals that influences mate recognition [3]–[6]. Thus, geographic divergence in sexual signals is regarded as an important factor in species formation, with bird song being a particular useful model [7]. The songs of oscine birds are unusual in that they serve a role in identifying conspecific mates, yet they are also culturally transmitted through vocal production learning [8], a phenomenon which is thought to accelerate allopatric speciation [9].

Darwin's finches of the Galápagos Islands have been inspiring evolutionary theory from the times of Darwin until today [10]–[13]. The adaptive radiation of this group of songbirds is one of the key examples of speciation and their study has revealed many important insights into evolutionary processes [e.g. 14]–[20]. The males of all Darwin's finches sing a single, structurally simple, and unvarying song throughout life that is culturally transmitted from one generation to the next [11], and it has been shown that females use these songs for species recognition and mate choice [21]. Thus, song divergence may constrain the mating of females to conspecifics and thus could potentially play a crucial role in speciation by promoting genetic isolation on secondary contact [19], [21], [22].

**Figure 1:**
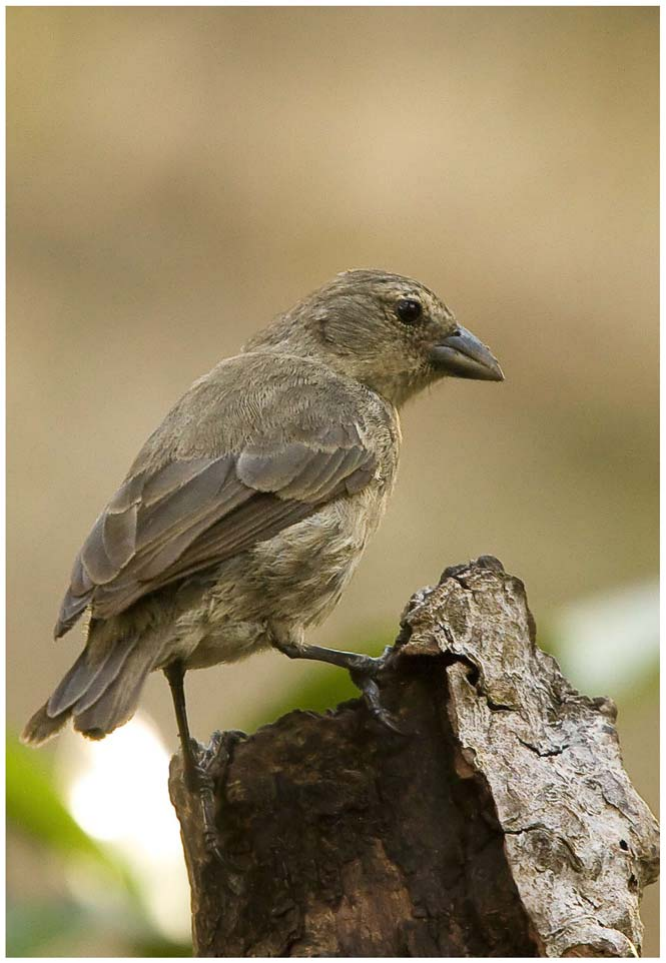
A mangrove finch, the rarest of Darwin's finches. The population of the Galapagos mangrove finch (*Camarhynchus heliobates*) has fallen to about 100 individuals, making it one of the world's most critically endangered birds. The species is now entirely confined to mangrove forests on the east and west coast of the island of Isabela. The main reasons for the decline in the mangrove finch are probably the impact of introduced pest species. The black rat (*Rattus rattus*) arrived in the Galápagos on pirate vessels perhaps as early as the 16th century, whereas feral cats (*Felis catus*), the smooth-billed ani (*Crotophaga ani*), two species of fire ants (*Wasmannia auropunctata* and *Solenopsis geminate*) and a parasitc fly (*Philornis downsi*) have been introduced by humans more recently. Photo by Michael Dvorak.

The mangrove finch *Camarhynchus heliobates* (Fig. 1) is not only the rarest of Darwin's finches, but one of the rarest birds in the world with an estimated population size of about 100 individuals [23]. Historically the species occurred on the Galápagos islands of Isabela and Fernandina but has now disappeared from the latter [23], [24]. To date, the surviving birds are confined to two geographically separated populations on Isabela, one on the west coast of the island and a second, very small population on the east coast [23], [25]. The habitat of this species is confined to small, disconnected patches of mangrove that are bordered by the sea on the one side and bare lava on the other. The two remnant mangrove finch populations are geographically separated by more than 70 km of barren lava desert and volcanic mountains. The presence of mangrove finches on the east coast of Isabela was discovered in 1900 [26], thus the two populations have been separated for at least 110 years.

During earlier surveys, between three and five singing males were observed on the east coast of Isabela [23], and the songs of these males appeared to sound different from those of the west coast [25]. Here, we report a systematic bioacoustic analysis of songs of the two populations. In addition, we tested with playback experiments whether mangrove finches discriminate between songs from the two localities, which would affect mate choice and thus reproductive isolation. To further investigate possible speciation in the mangrove finch, we also studied morphological and genetic divergence between the two remnant populations.

## Materials and Methods

### Ethics statement

All work was conducted in accordance with the “Guidelines for the Treatment of Animals in Behavioural Research and Teaching” published by the Association for the Study of Animal Behaviour, and with the appropriate licences and permissions by the Galápagos National Park Service and the Charles Darwin Foundation.

### Song recordings and acoustic analysis

Song recordings and playback experiments were carried out on the Island of Isabela (Galápagos, Ecuador) in February and March 2009. During an extensive survey of the east coast of Isabela in February 2009 we found only two males, which indicates a further population decline since the last survey in 2008 [23]. The songs of both eastern males were digitally recorded with a Marantz PMD 660 solid state recorder (sampling rate: 44.1 kHz) and a Sennheiser Me67 directional microphone. With the same equipment we recorded the songs of 20 of the estimated 40 remaining males of the western population. Some males were colour-banded (see below), and in addition three un-banded western individuals could be recognized by their unique plumage coloration. Previous observations of marked mangrove finches showed a strong site fidelity, within and between years. All the recordings were done within one week, and therefore, we are confident in identifying individual males based on the location of their territories in cases were un-banded birds were included in the analysis. Up to 46 (mean 16) of the highest quality song recordings of each bird were analysed using Avisoft-SASLAb Pro (R. Specht, Germany). Four acoustic parameters were measured from each song: song duration (s), mean syllable duration (s), syllable rate (Hz) and peak frequency (Hz), and then average values were calculated for each male. For the analysis of temporal parameters, we calculated spectrograms with a FFT-length of 256, which yielded a temporal resolution 5.8 ms. For the spectral analysis, the recordings were down sampled to 16 kHz, high-pass filtered (*f*_co_ = 0.4 kHz, Hamming window, 1024 coefficients), and measures were taken from spectrograms with a FFT of 1024, which yielded a spectral resolution of 16 Hz. Differences in acoustic song characteristic between the two populations were examined with Mann-Whitney U tests. Two-tailed *P* values were Bonferroni-Holm adjusted [27].

### Construction of playback stimuli

All digital editing of the playback stimuli was done with Avisoft SASLAbPro software. In total, we constructed 12 playback files each containing the song of a different male (10 males from the west coast, 2 males from the east coast). Each playback file had a total duration of 120 seconds including 26 songs. The songs of all mangrove finches consist of one syllable type that is repeated from one to four times with the majority of songs having three syllables (Fessl, unpublished data). To account for the varying numbers of syllables, we used three- and two-syllable songs in the playback, thus mimicking the natural singing style of the species. From each source male we chose the three-syllable song rendition with the highest recording quality, which was then band-pass filtered to remove background noise (*f*_L_ = 1.2 kHz, *f*_H_ = 4.6 kHz, Hamming window, 1024 coefficients), and normalized for amplitude. Each source song was used with its original three syllables and also with the last syllable deleted to yield a 2-syllable song. The source song was copied in ten groups of two 3-syllable songs (separated by 1.1 s silence) and two groups of two 3-syllable songs and one 2-syllble song (separated by 1.1 s silence) into a wav-file (sampling rate: 44.1 kHz, accuracy: 16 bit). The two song groups containing 2-syllable songs were always the third and the ninth group in the sequence. The resulting twelve song groups were evenly distributed over the entire duration of the sound file. None of the birds was tested with its own songs or songs recorded closer than 200 m to its territory in order to minimize the chance that subjects were familiar with the stimuli. Although the songs of the last two eastern males were tested several times, the playback of eastern songs was not pseudoreplicated because the two males comprise the entire known population of the eastern lineage.

### Playback procedure

In total, we tested 20 males from the west coast population. Each subject received two playbacks, one with local songs and one with songs from the east coast. Half of the birds were tested with the local songs first, the other half with the foreign songs. Playback experiments were carried out from 20 to 26 March 2009 between 0700 and 1500 hours. Thirteen males were tested on the same day with 30–70 minutes between the two treatments. For logistic reasons, the remaining seven males were tested with the second treatment one or two days after the first playback, but both experiments were always carried out at approx. the same time of day (±2 hours).

The playback stimuli were broadcast by a Marantz PMD 660 connected to a Logitech mm 28 loudspeaker. Upon spotting a singing male, we positioned the playback loudspeaker inside the bird's territory in approx. 1.7 m height. We aimed at placing the loudspeaker approx. 8 m from the test bird at the beginning of each playback, and most of the subjects received both treatments from this initial distance. In the remaining birds, it was not possible to place the loudspeaker in 8 m for one or both treatments, and the distance between bird and loudspeaker ranged between 6 and 18 m. In these cases, however, there was no systematic difference in the initial distance between subject and loudspeaker between the two treatments (Wilcoxon signed-ranks test: *T* ^-^ = 8.5, *n* = 7, *p* = 0.469). Each subject was observed for 2 minutes before, during and after the playback. During each of the three stages of the observation we continuously recorded the bird's vocalizations with a Sony WM-D6C recorder and a Sennheiser Me66 directional microphone. Because female songbirds are very unlikely to show strong responses to playbacks in the field [8], we specifically targeted only males in our experiment. However, any potential behaviours by females during the experiment were recorded in the same way as the for the male focus birds.

### Analysis of playback responses

From the 20 birds tested, seven were excluded from the analysis because they could not be observed during the entire period of 6 minutes for each of the two playback trials or because neighbouring males and/or females approached the playback loudspeaker.

Variables measured during the playback period included the change in song rate compared to pre playback baseline levels (# songs/min), latency to approach playback loudspeaker (s), minimum approach distance (m) and time within 5 m of the loudspeaker (s). For the post playback period we assessed the subject's time within 5 m of the loudspeaker (s) and the change in song rate compared to baseline levels (# songs/min). As these six response measures were highly correlated with each other, we conducted a principal component analysis (PCA) on the initial response variables. A Kaiser-Meyer-Olkin test and a Bartlett test indicated that the data set was suited for a data reduction through a PCA (KMO measure of sampling adequacy  = 0.715, Bartlett's test of sphericity: χ^2^_15_ = 90.1, *p*<0.001). The PCA yielded two principal components with an eigenvalue higher than 1 (Table 1). The individual PC factors were used as a composite measure of the birds' response to the playbacks (playback response scores). We compared individual PC1 and PC2 factors between the two playback treatments with Wilcoxon signed-ranks tests for matched-pairs based on exact *P* values.

**Table 1:** Principal component loadings for the playback response variables.

response variable	PC1 (52%)	PC2 (31%)
latency to approach	**−0.671**	0.634
minimum approach	**−0.860**	0.308
time within 5 m during playback	**0.847**	−0.271
time within 5 m after playback	**0.865**	0.152
song rate during playback	0.523	**0.781**
song rate after playback	0.468	**0.815**

Highest values for each PC factor are shown in bold, and the percentage of variation in the response variables explained by each PC factor is shown in parentheses.

### Genetic analyses

Blood from 33 mangrove finches of the western population was collected between 2006 and 2009 and from one eastern male in 2009. For the collection of genetic samples, the birds were caught with mist nets and a minimum amount of blood was taken with micropipettes following venipuncture of the alar vein. Blood samples were genotyped at 16 highly polymorphic microsatellite loci [28], [29]. Microsatellite analysis has been proved very useful in the study of phylogenetic relationships among Darwin's finches [30], [31] because variation in mitochondrial and nuclear DNA sequences is insufficient for resolving relationships within more closely related species of this group [32].

Standard laboratory protocols were used for DNA extraction and genotype determination. Specific protocols and primer sequences are available elsewhere [28]. Two independent rounds of DNA extraction and genotyping of the eastern bird produced 100% agreement across all loci. The analysis indicated that the small remnant eastern population contains unique genetic variation. Hybridization with the congener *Camarhynchus pallidus* can be ruled out since only one of the seven unique east coast alleles appears in our sample from Isabela (*n* = 18). It is also very distinct from other species sampled from Isabela [30], including *Camarhynchus parvulus* (*n* = 6), *Geospiza fuliginosa* (*n* = 13) and *Certhidea olivacea* (*n* = 26). The east coast bird's likelihood scores are well outside the range of values for birds within these species, and in each case lie 3–12 standard deviations below the mean. Finally, contamination from other species can be ruled out since these loci are highly specific to Darwin's finches [28].

### Body measurements

During status surveys which were conducted between 2006 and 2009 [23], B.F. took body measures and pictures from 31 males of the western population and from one male from the east coast (the same individual from which a genetic sample was collected). The birds were captured with mist nets and then colour banded. Upon capture, measures of bill size were recorded, as well as tarsus and wing length, and weight.

## Results

**Figure 2:**
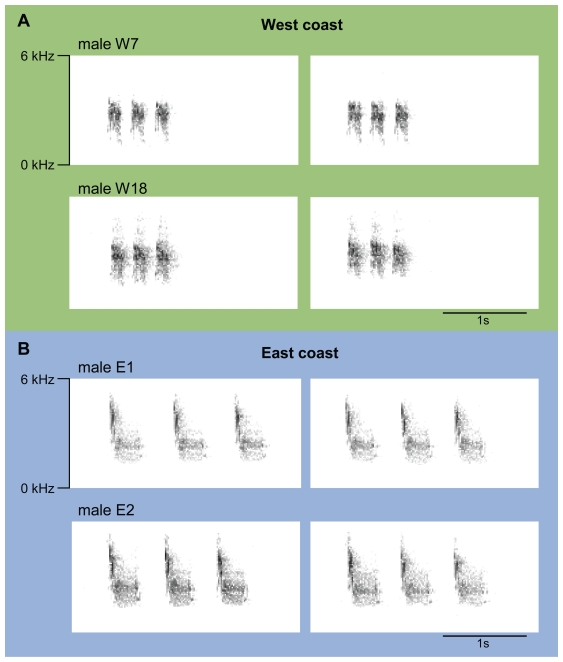
Song divergence between the two remnant mangrove finch populations. The figure illustrates the degree of structural song differences between populations and individuals as well as within individuals. **A** Spectrograms of two randomly selected song renditions from two exemplary males from the west coast population. All west coast songs looked similar to these. **B** Spectrograms of two randomly selected song renditions from the two eastern males.

The acoustic analysis yielded marked differences in song between the two mangrove finch populations (Fig. 2), and we found statistically significant differences in all measured song parameters (Table 2).

**Table 2:** Acoustic differences (means and ranges) between mangrove finch songs from the two remaining populations.

	population		between-site comparison	
acoustic parameter	west coast (*n* = 20 males)	east coast (*n* = 2 males)	Mann-Whitney *U* score	Uncorrected *p* value
song duration (s)	0.78 (0.69–1.06)	1.12 (1.02–1.23)	1.0	0.013*
syllable duration (ms)	154 (144–173)	386 (383–389)	0.0	0.004*
syllable rate (Hz)	4.2 (3.8–4.3)	1.9 (1.8–2.0)	0.0	0.009*
peak frequency (kHz)	2.98 (2.74–3.20)	3.60 (3.53–3.67)	0.0	0.009*

*Statistically significant at *p*<0.05 after Bonferroni-Holm correction.

**Figure 3:**
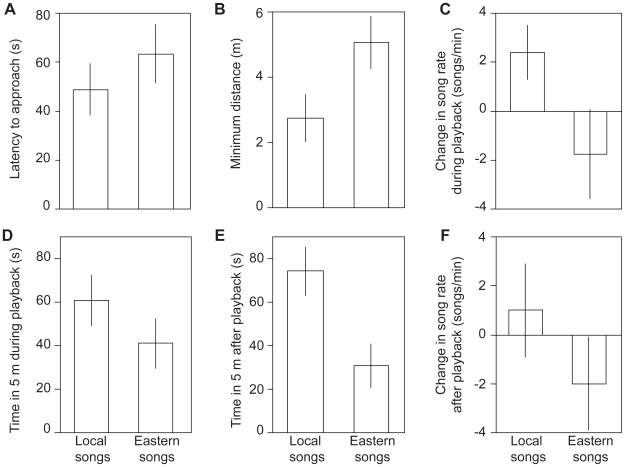
Responses of territorial mangrove finches from the west coast of Isabela to playback of local songs and songs from the east coast population, across six raw response variables (A–F). Means ± SE are shown, *n* = 13 males. Statistically significant differences between the two playback treatments were found for the song and approach parameters during the playback and the distance parameter after the playback (see text).

Moreover, we determined that the population differences in the songs were meaningful to the mangrove finches, as territorial males exhibited strong discrimination of the two local song variants. Principal component analysis revealed that males responded significantly stronger in response to the playback of local songs than to the songs from the other population according to PC1 (mean ± SD response: local songs  = 0.411±0.895; eastern songs  = −0.411±0.957; *T* ^-^ = 12, *n* = 13, *p* = 0.017), but not according to PC2 (local songs  = 0.134±0.839; eastern songs  = −0.134±1.158; *T* ^-^ = 35, *n* = 13, *p* = 0.497). PC1 explained the majority of the variation in the raw response parameters. An examination of the single response variables (Fig. 3) showed that the birds' stronger response to local songs was mainly due to a higher increase in song rate during the playback (*T* ^-^ = 0, *n* = 13, uncorrected *p* = 0.001, significance retained after Bonferroni-Holm correction), a closer approach to the loudspeaker (*T* ^+^ = 10, *n* = 13, uncorrected *p* = 0.021), and more time spent in the vicinity of the playback loudspeaker after the playback (*T* ^-^ = 11, *n* = 13, uncorrected *p* = 0.013). By contrast, no statistical differences were found in the latency to approach (*T* ^+^ = 33, *n* = 13, uncorrected *p* = 0.404), time spent within 5 m of the loudspeaker during the playback (*T* ^+^ = 26.5, *n* = 13, uncorrected *p* = 0.349) and the song rate after the playback (*T* ^-^ = 25, *n* = 13, uncorrected *p* = 0.161).

Female responses to the playbacks were rare, and in the majority of cases no female was observed in a males' territory at all. However, of the five cases in which females approached the loudspeaker four occurred during the playback of local songs from the western population and only one occurred during the playback of foreign songs.

The notion of two separate mangrove finch lineages on the east and west coast of Isabela is supported by additional genetic and morphological differences: the captured east coast male's breast had more pronounced dark streaks than was observed in any west coast male, and it also had a smaller beak gape than all 31 males that have been captured and measured on Isabela (east coast  = 8.25 mm, west coast  = 8.70–10.35 mm). When compared to 33 genotypes from the western population of *C. heliobates*, the eastern bird revealed seven unique alleles. It was homozygous for two of these unique alleles at two different loci, while only two other singleton alleles were found in the western population. The observed heterozygosity at the 14 autosomal loci for the eastern bird (43%) was slightly higher than the average for the western population (36%). Assignment tests [33] showed likelihood values of the eastern bird (−22.2) are well below the range of values for *C. heliobates* (mean±SD: −6.4±1.3).

## Discussion

We found marked acoustic differences in song between the two remnant populations of the mangrove finch. The observed acoustic divergence was as big as or even bigger than differences in song between different species of Darwin's finches (c.f. [34]). Territorial males at the west coast of Isabela responded more strongly to playback of local songs than to playback of songs from the eastern population, particularly in terms of song rate, nearest approach and time spent in the vicinity of the loudspeaker after the playback. The validity of the playback results is confirmed by comparison with earlier playback studies with Darwin's finches [22], [35]–[37]. Territorial males in these studies as well as in ours showed similar responses to playback in terms of song rate and nearest approach. Thus, the results of our playback experiment suggest that species recognition in mangrove finches is impaired by the song divergence between the two remnant populations, which will affect sexual selection by male-male competition upon secondary contact.

Our study is the first evidence of highly divergent genetic differentiation within a single island population of Darwin's finches and it is clearly associated with discontinuities in suitable habitat, as well as divergence in sexual signals. In terms of reproductive isolation, the crucial question is whether females discriminate between the local song variants in a similar way as the males did. Typically, females are much less responsive to song playback than males, and most field studies on species recognition have looked at responses of territorial males [8]. Just as in a prior study on another species of Darwin's finch, the sharp-beaked ground finch *Geospiza difficilis* [36], female mangrove finches in this study were very rarely observed to respond to the playback. However, of the five observed cases, four females came near the loudspeaker during the playback of local songs and only one female approached while a foreign song was broadcast. Thus, assuming that female mangrove finches exercise similar discrimination of songs by locality as males, it might well be that the two mangrove finch populations are already reproductively isolated and could be regarded as two separate species. If so, the eastern species will face imminent extinction because it has an estimated population size of only 5–10 individuals [23].

It has been shown that local variants in bird song can be adapted to the environmental acoustics of different habitats [38]. Both finch populations occur in mangrove forests, but the mangrove trees on the west coast were on average more than two times higher than on the east coast, and the canopy was also significantly less closed in the western forests [25]. However, these habitat differences are rather small compared to those that have been found important in previous studies [e.g. 39]–[41], and it remains to be shown whether they can be accounted for the divergence observed in mangrove finch song. Alternatively, and more likely, the song differences may be the outcome of random processes coupled with cultural transmission through individual learning of mating signals [9]. The two mangrove finch populations are geographically separated by 70 km of barren lava desert and volcanic mountain, which is likely to have promoted the cultural evolution of different local song dialects. Initially, the population differences in the mating signal may be due to a founding effect that was then maintained by vocal tradition. Another possibility is that the divergent trajectories of evolution among the isolated populations may reflect a more gradual cultural drift of song characteristics over longer times. In any case, our study suggests that crucial parts of the speciation process can occur in complete allopatry, without any sympatric phase at all.

Our findings not only provide strong support for the central role of sexual signals during speciation, but at the same time they also have implications for conservation. Critically endangered species are often assumed to be relatively homogeneous. Our results show, however, that even small numbers of individuals may harbour reproductive incompatibilities that have accumulated through isolation and divergence at small spatial scales. If individuals from the two populations are not able to successfully reproduce with each other, then the scope of conservations actions such as captive breeding or translocation of birds will be limited.
